# To Dr. Bradley

**Published:** 1808-07

**Authors:** W. Blair

**Affiliations:** Great Russel Street


### To Dr. BRADLEY.

Dear Sir,

I Am sorry it should be necessary for me to complain of the manner in which you have expressed yourself, on certain passages of my "Hints to Parliament" (see pp. 561 and 562, No. 112, Med. and Phys. Journ.) After quoting a few lines from my book, in which I censure Dr. Walker, and those "*few individuals*" who countenanced his proceedings against the Jennerian Society, you say, "the rancour, virulence, and falsehood" of my language has not been exceeded by "Birch, Rowley, or Moseley." Now, Sir, it is very obvious to me that you have quite mistaken the import of my words, and considered them as applicable to "the founders of the London (Vaccine) Institution *as a body*," rather than to the *few* persons who have acted as Dr. Walker's abettors. I had no design to reflect on those persons in the mass, and have admitted that "*many*" of them are "very excellent and benevolent:" though they may may have been deceived by Dr. Walker's contrivances, and unsuspectingly encouraged his illiberal opposition. You have yourself condemned his conduct in some respects, and I am told that he has used even your name beyond what you authorized: surely, then, you will not deny that he might possibly have done many other things of which you were ignorant, and of which he has been publicly accused by the Directors of the Royal Jennerian Society, as well as by several individuals of high rank who do not belong to this Institution.

I felt it due to my own character, thus to offer some explanation on the above subject; but I by no means desire to enlarge, with the view of criminating Dr. W.—And, as to the other point, concerning which you have considered my observations too personal, I can only say, that I very much regret to have let a single passage appear in my book, which can admit of that construction. But, I think it fair to avail myself of the apology I made (at p. 41) in the contemplation of such a possible event; *viz.* "Should a *verbum ardens* escape me during the hurry and rapidity of my writing, too like the envenomed acrimony or bitter calumnies of the author himself (Mr. Birch), you will expunge such words from this letter, and impute them to mere inadvertency." Besides this, I had distinctly expressed the great unwillingness with which I commented on the practice of the "two respectable gentlemen at the Small-pox Hospital."

You will oblige me by giving this exculpatory note a place in the next Number of the Med. and Phys. Journal, lest the severity of your criticism should leave an impression on your readers beyond the measure of strict justice.

I am &c.

W. BLAIR.

*Great Russel Street, June 18, 1808.*

